# Ocular toxicities of targeted therapies and immunotherapies in hematologic malignancies

**DOI:** 10.3389/fonc.2025.1691518

**Published:** 2026-01-06

**Authors:** Mohammad S. Aqil, Yzen Al-Marrawi, Marcus Yaldo, Ahmad Abu-Mahfouz, Lavi Singh, Swathi Gopishetty, Precious Idogun, Daniel Ezekwudo, Ishmael Jaiyesimi, Adam J. Weiner

**Affiliations:** 1 Oakland University William Beaumont School of Medicine, Rochester, MI, United States; 2 Hematology Oncology Department, Corewell Health, Royal Oak, MI, United States; 3 Hematology Oncology Department, Florida Cancer Specialists & Research Institute, Fort Myers, FL, United States; 4 Beaumont Eye Institute, Corewell Health, Royal Oak, MI, United States; 5 Associated Retinal Consultants, P.C., Royal Oak, MI, United States

**Keywords:** hematologic malignancies, targeted therapies and immunotherapies, ocular toxicities, neuro-ophthalmic complications, pharmacovigilance

## Abstract

Targeted therapies and immune-based treatments have transformed the management of hematologic malignancies. However, these agents can also result in unintended ocular adverse effects. These toxicities are often underrecognized but may significantly affect patient quality of life and therapeutic decision-making. A comprehensive understanding of these effects is essential for interdisciplinary management. This review synthesizes the current evidence regarding ocular toxicities associated with modern targeted and immune therapies used in hematologic cancers. Data were drawn from case reports, clinical trials, observational studies, and pharmacovigilance databases. Ocular side effects were reported across all major therapy classes, including cell therapies, kinase inhibitors, immune checkpoint inhibitors, monoclonal antibodies, antibody–drug conjugates, and proteasome inhibitors. Chimeric antigen receptor T-cell therapies commonly induce neuro-ophthalmic symptoms such as photophobia and visual disturbances, frequently in association with neurotoxicity syndromes. Tyrosine kinase inhibitors were associated with a range of effects, including periorbital edema, uveitis, and retinal vascular complications. Immune checkpoint inhibitors caused inflammatory eye diseases such as uveitis, optic neuritis, and ocular myasthenia, consistent with immune-related adverse events. Certain antibody–drug conjugates, particularly those used in multiple myeloma, produced high rates of ocular surface disease that required dose modifications. While many adverse effects were reversible, some caused vision-threatening complications that required prompt ophthalmologic intervention. Pediatric-specific data were sparse, and long-term ocular outcomes remain poorly defined. Ocular toxicities from modern hematologic cancer therapies span a broad clinical spectrum and vary by drug class. Increased awareness of these complications among oncologists and ophthalmologists can support earlier detection and treatment. Accurate clinical descriptions of ocular adverse events and effective management recommendations in these settings will help improve patient outcomes and their quality of life.

## Background

Targeted therapies and immunotherapies have revolutionized the treatment of hematologic malignancies over the past 15 years. Chimeric antigen receptor T-cell (CAR T-cell) therapies, tyrosine kinase inhibitors (TKIs), immune checkpoint inhibitors (ICIs), and other novel agents (e.g. bispecific antibodies, monoclonal antibodies, antibody-drug conjugates, and small-molecule inhibitors) have greatly improved outcomes in leukemias, lymphomas, and myeloma (1, 2). In parallel, bispecific T-cell engager (BiTE) therapies, such as blinatumomab, have seen growing application in solid tumors, though many of these uses remain investigational (3). These therapeutic advances, however, are accompanied by a spectrum of unique adverse effects, including ocular toxicities (4). Such complications ranging from mild dry eye to severe, vision-threatening pathology, are increasingly recognized, yet often underreported (5). These adverse events can significantly impact patients’ quality of life and may limit therapy if severe (6). To date, information regarding ocular side effects in hematologic-oncology patients has been scattered across case reports, reviews, clinical trials, and pharmacovigilance databases.

This review presents a comprehensive analysis of ocular toxicities associated with contemporary therapies for hematologic cancers. We examine CAR T-cell therapies, TKIs, ICIs, and other targeted agents across both adult and pediatric populations worldwide. Key topics include incidence, clinical presentation, proposed mechanisms, time to onset, management strategies, and patient outcomes. This review summarizes published evidence and describes interventions reported by treating clinicians; it does not provide clinical practice guidelines or prescriptive recommendations. Additionally, we identify current knowledge gaps and outline priorities for future investigation. By consolidating this information, clinicians in both oncology and ophthalmology can better anticipate, recognize, and manage therapy-related ocular complications while understanding that the management approaches discussed reflect reported practice rather than formal guidance.

## Methods

A comprehensive literature search was performed across PubMed, Embase, and Web of Science for records published from 1 January 2004 through 31 March 2025. Strategies combined controlled vocabulary and keywords for ocular outcomes (i.e., eye, ophthalm*, dry eye, keratitis, keratopathy, uveitis, scleritis, uveal effusion, optic neuritis, optic neuropathy, retinitis, retinopathy, macular edema, retinal detachment, zoster) with therapy and drug-class terms across relevant agents (i.e., CAR T-cell therapies, immune checkpoint inhibitors, tyrosine kinase inhibitor, antibody-drug conjugates, proteasome inhibitors). To avoid missing therapy-centric reports, we ran query blocks with and without disease-name terms; “leukemia,” “lymphoma,” and “myeloma” were not required in all searches, and hematologic eligibility was confirmed during screening. Reference lists of pertinent articles and key reviews were also checked to identify additional studies.

Eligible studies included peer-reviewed clinical trials, observational studies, case series, and case reports that reported ocular adverse events associated with CAR T-cell therapies, TKIs, ICIs, or other targeted agents in patients with hematologic malignancies. Reviews were used for citation chasing and background only and were not counted among included studies. Both adult and pediatric populations were considered. Exclusion criteria encompassed studies that did not report ocular outcomes. The review was conducted in accordance with systematic review principles; although elements of the PRISMA framework were employed, full adherence to all PRISMA criteria was not implemented. Five reviewers independently conducted title and abstract screening, full-text eligibility assessment, and data extraction. Extracted data included incidence, type, onset, management, and outcomes of ocular toxicities. Discrepancies were resolved through discussion and consensus.

Due to significant heterogeneity in study designs, populations, and outcome measures, data synthesis was primarily qualitative. Where available, incidence rates from large-scale clinical trials and pharmacovigilance sources were reported and supplemented by descriptive findings from smaller studies and case reports. Although this review prioritizes FDA-approved therapies, data on investigational agents were included when clinically relevant. Results are presented by therapeutic class, with detailed characterization of the associated ocular toxicities. The limited availability of robust data, particularly in pediatric cohorts, is acknowledged as a limitation and is addressed in the discussion regarding the overall strength and consistency of the evidence.

## Results

### Overview of included studies

Across databases and citation chasing, the search retrieved 652 records. After removing 78 duplicates, 574 titles and abstracts were screened. We assessed 264 full texts for eligibility. Fifty-seven studies were included in the qualitative synthesis, and thirty-eight reported extractable incidence data. Most data on ocular toxicities come from retrospective analyses and pharmacovigilance reports, with a few prospective observations (particularly for newer agents that mandated ocular monitoring). The vast majority of reports involve adult patients; pediatric-specific data were sparse, largely limited to CAR T-cell therapy in acute lymphoblastic leukemia (ALL). Key findings are summarized by therapy modality below. Additionally, **Table 1** provides a consolidated overview of the major therapeutic classes and their characteristic ophthalmic complications.

**Table 1:** Targeted and immunotherapies used in hematologic malignancies and their characteristic ophthalmic complications.

Class	Representative agents	Characteristic ophthalmic complications
CAR T-cell therapy	Tisagenlecleucel, axicabtagene ciloleucel	Photophobia, blurred vision, papilledema, optic neuritis, uveitis; infectious events such as HZO or viral retinitis; most improve with control of CRS/ICANS and steroids (7)
Bispecific T-cell engagers/antibodies	Blinatumomab, teclistamab, mosunetuzumab	No consistent ocular syndrome reported; complaints rare, usually with systemic toxicity (8–11)
Immune checkpoint inhibitors (PD-1/PD-L1, CTLA-4)	Nivolumab, pembrolizumab, ipilimumab	Uveitis (VKH-like), serous/exudative retinal detachment, keratitis, scleritis, optic neuritis, autoimmune retinopathies, ocular myasthenia. Most resolve with corticosteroids (12–14)
Tyrosine kinase inhibitors		
*BCR-ABL inhibitors*	Imatinib, dasatinib, nilotinib, bosutinib, ponatinib	Periorbital edema; conjunctival hemorrhage; macular or optic disc edema; ponatinib linked with retinal vascular events (6, 15)
*BTK inhibitors*	Ibrutinib	Dry eye, blurred vision, uveitis, cystoid macular edema, ocular bleeding (16–18)
*JAK inhibitors*	Ruxolitinib	Herpes zoster ophthalmicus with keratitis or uveitis (19)
*FLT3 inhibitors*	Midostaurin, gilteritinib	Eyelid edema (~3%); rare blurred vision or photophobia (midotaurin) (20). Low rates, including blurred vision, dry eye disease, and retinal hemorrhage (~6–7% each); rare differentiation syndrome which can include visual symptoms (gilteritinib) (21)
Antibody–drug conjugates	Belantamab mafodotin	Corneal epithelial toxicity/keratopathy (whorl-like epitheliopathy, decreased vision); often reversible with dose holds and ophthalmic monitoring (22)
Monoclonal antibodies	Rituximab, alemtuzumab, daratumumab	Serum sickness–like reaction (rare optic neuritis), thyroid eye disease (alemtuzumab) (23), transient choroidal effusion (daratumumab (24))
Proteasome inhibitors	Bortezomib, carfilzomib, ixazomib	Eyelid disorders, especially chalazia and blepharitis (bortezomib); blurred vision with ixazomib (25–28)
Immunomodulatory drugs (IMiDs)	Thalidomide, lenalidomide, pomalidomide	Blurred vision (rare), papilledema at high doses; generally mild and reversible (29)

This table lists major drug classes, representative agents, and typical ocular events reported for each. Adverse events range from inflammatory disorders (uveitis, keratitis, scleritis), neuro-ophthalmic findings (optic neuritis, papilledema), and retinal pathology (cystoid macular edema, serous or exudative retinal detachment) to infection-related complications (herpes zoster ophthalmicus, viral retinitis) and ocular surface or eyelid disease (eg, chalazia and blepharitis with proteasome inhibitors). Tyrosine kinase inhibitors are summarized here and are discussed in greater detail by subclass in **Table 2**.

### Ocular toxicity with CAR T-cell and BiTe therapies

CAR T-cell therapies (such as anti-CD19 CAR Ts for acute lymphoblastic leukemia and large B-cell lymphoma, and BCMA-directed CAR Ts for multiple myeloma) have well-known systemic toxicities like cytokine release syndrome (CRS) and immune effector cell-associated neurotoxicity syndrome (ICANS). Ocular adverse events were not highlighted in initial clinical trials, but emerging pharmacovigilance data and case series indicate that a subset of patients experience ocular complications. A recent FDA adverse event database analysis identified 53 ocular adverse events from 2017 to 2023 linked to the six FDA approved CAR T products (5). Although overall reporting rates were low (~2% of all CAR T adverse events in the database were ocular) (7), the signal for ocular toxicity was significant and not reflected in product labeling (8). A separate single-center series found that 11 of 66 adult lymphoma patients (17%) developed new ocular symptoms (see below) after CAR T infusion (7), suggesting ocular issues may be more common if actively monitored. Pediatric CAR T recipients may have similar toxicities, though specific pediatric-focused reports are lacking.

#### Incidence and clinical spectrum

The ocular events reported with CAR T therapy range from transient mild symptoms to serious complications. The most frequently reported issues include blurred or altered vision, photopsia (perceived flashes of light) and floaters, pupillary dilation (mydriasis) with sluggish light reflex, and photophobia. In the FDA database study, tisagenlecleucel accounted for about 60% of reported ocular events and axicabtagene ciloleucel for ~39% (7). The most common events with tisagenlecleucel were nonspecific vision changes or impairment, mydriasis, papilledema secondary to ICANS, and photophobia (7). These findings align with the clinical observation that many CAR T-related ocular toxicities are neuro-ophthalmic, for example, papilledema and vision changes often occur in the context of ICANS (where raised intracranial pressure or direct neurotoxicity may affect the optic nerve) (7). In the single-center series, 10 of the 66 patients developed new ocular issues a median of 1 month post-CAR T: two had new onset photopsia/floaters suggestive of vitritis or retinal involvement, two experienced reactivation of herpes zoster ophthalmicus (HZO) with keratitis, and one had an exacerbation of preexisting ocular graft-versus-host disease (7). Isolated cases of uveitis and optic neuritis have also been described following CAR T-cell therapy. However, in heavily pretreated patients, distinguishing treatment-related uveitis from infectious, autoimmune, or drug-induced etiologies can be challenging.

#### Proposed mechanisms

Ocular toxicities from CAR T are thought to be largely immune-mediated or indirect. Many reported ocular events coincide with CRS or ICANS, suggesting a pathophysiologic link (30). The intense cytokine release (specifically IL-6 and IFN-γ) in CRS could disrupt the blood–ocular barriers, leading to inflammation (e.g. uveitis) or optic nerve edema. Similarly, ICANS-related cerebral edema can manifest as papilledema or cranial nerve palsies affecting vision (7). Some CAR T ocular effects may represent neurologic toxicity presenting with visual symptoms (for instance, cortical visual disturbances). Immune dysregulation may also trigger reactivation of latent infections in the eye, e.g. HZO as seen in some patients, likely due to immunosuppression from lymphodepleting chemotherapy and CAR T-cell effects (7). HZO can lead to keratitis, uveitis, and chronic ocular surface pain if not promptly treated. Unlike classic drug toxicity, CAR T-cells themselves do not target eye structures (the antigens like CD19 or BCMA are not expressed in ocular tissues), so direct “on-target/off tumor” attack on the eye is not expected. Instead, systemic inflammation, blood–brain/ocular barrier permeability changes, and immune effector cell trafficking are implicated. One hypothesis is that CAR T cell activation in the CNS or meninges could cause a lymphocytic uveitis or vasculitis in ocular tissues; another is that the high cytokine levels (IL-6, IFNγ, etc.) disturb the ocular microenvironment (30). Further research is needed to clarify these mechanisms, as none are definitively proven.

#### Timing

Ocular toxicities associated with CAR T-cell therapy have most commonly been observed during the acute period following infusion. In reported cases, symptom onset typically occurred within days to several weeks post-infusion and often coincided with the development of CRS or ICANS. In a case series by Mumtaz et al., the median time from CAR T-cell infusion to the onset of ocular symptoms was reported to be 25 days (7). Symptoms like photophobia, vision blur, or floaters often arose during the hospitalization or soon after discharge. Infectious complications like HZO tended to occur a bit later (several weeks post infusion) in patients with prolonged cytopenias or steroid treatment. To date, late onset ocular issues (months to years later) have not been prominently reported with CAR T, although delayed effects cannot be ruled out given the novelty of these therapies. Long-term follow-up of CAR T survivors (including pediatric ALL survivors) for ocular health has not been well described and represents a gap in knowledge.

#### Outcomes and management

Most CAR T-related ocular toxicities reported were reversible with appropriate management. In the FAERS analysis, some ocular events had serious outcomes (including a few with fatal outcomes) (30). Encouragingly, in published case series patients generally recovered normal or near-normal vision after treatment of the underlying CAR T toxicity. Management centers on addressing the systemic syndrome: prompt therapy for CRS/ICANS (e.g. tocilizumab, corticosteroids) often led to resolution of associated ocular findings like papilledema and photophobia. Ophthalmology consultation is advised for any visual complaints after CAR T infusion (7). Specific interventions have included high-dose corticosteroids for immune-mediated uveitis or optic neuritis, intravitreal antivirals for viral retinitis, and topical or systemic antivirals for HZO keratitis, alongside supportive measures (lubricating drops for dryness, etc.). While rare, viral retinitis is a serious and potentially blinding conditions, and outcomes remain poor even with prompt intravitreal and systemic antiviral therapy due to the high risk of retinal detachment. In the case of HZO, patients were treated with systemic acyclovir and topical steroid drops, with good reported outcomes; however, without appropriate antiviral prophylaxis, HZO may recur after treatment discontinuation (7). Permanent vision loss following CAR T-cell therapy appears to be exceedingly rare in the published literature. One reported case involved a patient who developed irreversible cortical blindness secondary to ICANS, representing a neurologic complication rather than direct ocular injury. Overall, early recognition and appropriate management of ocular symptoms have been associated with favorable outcomes and prevention of long-term sequelae. Notably, ocular adverse events have not necessitated discontinuation of CAR T-cell therapy in reported cases, with clinical management directed primarily toward concurrent neurotoxicity.

#### Bispecific T-cell engagers and bispecific antibodies (e.g. blinatumomab, teclistamab, mosunetuzumab)

These novel immunotherapies recruit T-cells to kill cancer cells by dual-binding (e.g. blinatumomab binds CD19 on B-cells and CD3 on T-cells) (31). Their side effects often resemble a tempered version of CAR T toxicities – cytokine release syndrome and neurotoxicity are primary concerns (32). In terms of ocular toxicity, no unique syndrome has been attributed to bispecific engagers so far (33). Blinatumomab’s clinical trials in acute lymphoblastic leukemia did not report significant eye-specific adverse events; however, cases of neurotoxicity with blinatumomab can include visual disturbances (blurred vision, hallucinations, etc.) as part of encephalopathy. These are neurological in origin rather than from direct eye injury (8). Teclistamab (a BCMA bispecific for myeloma) and mosunetuzumab (CD20xCD3 for lymphoma) similarly have no known direct ocular toxicities apart from rare occurrences of transient blurred vision or dizziness during cytokine release fevers (9, 10). Any such symptoms typically resolve once CRS is managed (9). Given their mechanism, it is possible that severe cytokine release could cause endothelial leakage in the eye (e.g. macular edema or retinal vascular leakage), but this hasn’t been specifically documented (11). Overall, bispecific antibodies have not shown a distinct ocular toxicity profile to date, though vigilance is still necessary given their increasing use.

#### Clinical recommendations and gaps

Given these findings, patients receiving CAR T-cell therapy should be educated to report visual changes promptly. A baseline eye examination is most important for those with known preexisting ocular conditions (e.g., diabetic retinopathy, glaucoma, prior ocular surgery, history of uveitis, or previous ocular infections such as HZO), though it is not yet standard practice. Furthermore, patients should be counseled in advance to maintain a low threshold for reporting any new visual disturbances or ocular symptoms. During the high-risk period (first 4–6 weeks post-infusion), oncology teams should also maintain a low threshold to involve ophthalmologists if patients report eye pain, changes in vision, or other ocular symptoms (30). This interdisciplinary approach supports early recognition and management of ocular complications. In addition to clinical examination, ophthalmologists may utilize fluorescein angiography to detect early endothelial vascular leakage or subclinical inflammatory changes. Particular attention should be given to pediatric CAR T recipients, who may have difficulty expressing visual changes. Moreover, extended ocular surveillance is warranted to assess for delayed autoimmune manifestations that could arise from persistent immune activation. Thus far, ocular toxicities are noteworthy but not extensively documented, underscoring the need for ongoing pharmacovigilance and reporting.

### Ocular toxicity with tyrosine kinase inhibitors and small molecule inhibitors

TKIs are widely utilized in the treatment of hematologic malignancies. Examples include BCR-ABL inhibitors for CML and ALL, such as imatinib, dasatinib, nilotinib, bosutinib, and ponatinib; Bruton tyrosine kinase inhibitors for CLL and lymphoma, including ibrutinib, acalabrutinib, and zanubrutinib; JAK inhibitors for myeloproliferative neoplasms, such as ruxolitinib and fedratinib; and FLT3 inhibitors for AML, such as midostaurin and gilteritinib. While TKIs exert their therapeutic effects by selectively inhibiting dysregulated signaling pathways, many also exhibit off-target activity on receptors expressed in ocular tissues or induce systemic effects that may impact ocular health (6). Ocular side effects of TKIs, while generally not life-threatening, are not uncommon and can affect multiple ocular structures. Incidence varies by agent, but certain class-specific patterns are recognized (as summarized in **Table 2**) (11).

**Table 2:** Ocular adverse events associated with select tyrosine kinase inhibitors (TKIs) and other small molecule inhibitors.

TKI/Small molecule inhibitor	PMT	Cancer indication	Adverse ocular events
Imatinib	BCR-ABL, cKIT, PDGFR	CML, GIST, ALL	Periorbital edema, conjunctival hemorrhage, blurred vision, dry eye, macular edema, optic disc edema (6, 34)
Dasatinib	BCR-ABL, SRC	CML, ALL	Periorbital edema, macular edema, optic disc edema (6, 34)
Nilotinib	BCR-ABL	CML	Periorbital edema, eyelid redness, chorioretinopathy, visual disturbances (6, 34).
Ponatinib	BCR-ABL, VEGFR, FGFR	CML	Retinal vein occlusion, macular edema, retinal hemorrhage, optic disc edema (15, 35)
Ibrutinib	BTK	CLL, MCL	Blurred vision, dry eye, uveitis, cystoid macular edema, ocular bleeding (34)
Ruxolitinib	JAK1/2	Myelofibrosis, PV	Herpes zoster ophthalmicus with keratitis and uveitis (19)
Dabrafenib	BRAF	Melanoma	Uveitis, cystoid macular edema, uveal effusion, serous/exudative retinal detachment (36)
Vemurafenib	BRAF	Melanoma	Uveitis, conjunctivitis, dry eye, VKH-like syndrome (37)
Midostaurin	FLT3	AML	Eyelid edema (~3%); rare blurred vision or photophobia; no consistent structural ocular signal in AML trials (20).
Gilteritinib	FLT3	AML	Visual changes reported at low rates, including blurred vision, dry eye disease, and retinal hemorrhage (~6–7% each); rare differentiation syndrome which can include visual symptoms (21).
Erdafitinib	FGFR1–4	Metastatic urothelial carcinoma	Central serous retinopathy, RPED, dry eye, PUK (38)
Crizotinib	ALK, ROS1	NSCLC	Photopsia, blurred vision, vitreous floaters (39)
Venetoclax	BCL2 inhibitor	CLL, AML	Blurred vision (transient, non-pathologic) (40)

Representative agents are listed with their primary molecular targets (PMT), approved cancer indications, and characteristic ocular toxicities. Reported events span benign, common findings (for example, periorbital edema with BCR-ABL inhibitors) to vision-threatening complications (for example, retinal vein occlusion with ponatinib, uveitis/CME with ibrutinib). Incidence and severity vary by agent and mechanism of action. Agents used outside hematologic malignancies (for example, BRAF or ALK/ROS1 inhibitors) are included where they illustrate class-specific ocular signals relevant to oncology practice.

BCR-ABL TKIs (Imatinib, Dasatinib, Nilotinib, Ibrutnib, Bosutinib, Ponatinib): These are central to CML treatment and have well-documented ocular effects. Periorbital edema (swelling around the eyes) is the most frequent issue, especially with imatinib: it occurs in up to ~60% of patients on imatinib long term (6). This often presents as puffiness of the eyelids and can lead to epiphora (excess tearing) due to nasolacrimal duct compression (6). Imatinib is also associated with conjunctival hemorrhages in ~2–11% of patients, thought to result from inhibition of c-KIT on conjunctival mast cells (6). Blurred vision and dry eye symptoms are reported in ~1–10% (34). Ibrutinib has been anecdotally associated with cataracts and retinal artery occlusion; however, a clinical trial involving over 500 patients found no increased incidence of cataracts compared to the general population. While rare, these ocular events should still be considered potential complications (41). Rarely, imatinib and dasatinib have caused optic disc edema and macular edema, sometimes in conjunction with fluid retention syndromes (34). These findings may reflect endothelial barrier dysfunction and vascular leakage, consistent with other TKI-associated ocular events. Ponatinib, a later-generation TKI, has unique vascular toxicity: in clinical trials, retinal vein occlusion, retinal hemorrhage, and macular edema occurred in ~2–3% of ponatinib-treated CML patients (15). Ponatinib’s package insert carries a warning about these retinal events, and cases of optic disc edema from presumed ponatinib-induced hypertension have been observed (35). Nilotinib commonly causes periorbital edema and eyelid redness (6), and infrequently can precipitate central serous retinopathy (CSR) or visual disturbances (34). Among ocular adverse events, periorbital edema represents a non–vision-threatening, typically benign class effect (most common with imatinib), whereas retinal vascular events such as arterial or venous occlusion are vision-threatening and typically irreversible (34). Vascular side effects (embolism or thrombosis) from TKIs like ponatinib or nilotinib may indirectly lead to ocular ischemic events, though these remain exceedingly rare (34).

#### Bruton TKIs (ibrutinib and next-generation agents)

Ibrutinib (for CLL, mantle cell lymphoma, etc.) has been linked with a variety of ocular side effects. Large trials reported blurred vision in about 10–13% of patients and dry eye in ~17% (16). Increased tearing and reduced visual acuity were also noted in ~10% each (16). These symptoms are typically mild, however, there are cases of ibrutinib-induced uveitis (17). Patients on ibrutinib have presented with anterior uveitis and vitritis within weeks to months of starting therapy (18). Some developed cystoid macular edema as part of the uveitis syndrome, potentially reflecting endothelial barrier disruption similar to that described with other targeted agents (17). Ibrutinib’s kinase profile (hitting off-target kinases in B-cells and perhaps ocular immune cells) is suspected in precipitating these inflammatory events. Fortunately, uveitis usually responds to topical steroids and holding the drug, and patients have been able to continue ibrutinib with added ocular therapy in some cases (17). Ibrutinib also impairs platelet function, so cases of ocular bleeding have occurred: e.g. a spontaneous hyphemia was reported in a patient, and another case had a branch retinal artery occlusion attributed to ibrutinib-associated thrombosis (17). Second-generation BTK inhibitors (acalabrutinib, etc.) might have similar but less frequent ocular issues, though less data are published. Overall, dry eye and blurred vision are relatively common with this class, while uveitis is an uncommon but noteworthy immune side effect.

JAK2 Inhibitors (Ruxolitinib and others) – JAK inhibitors in myelofibrosis and polycythemia vera have potent immunosuppressive effects. While direct ocular toxicity is not prominent, the main concern is opportunistic infections involving the eye due to immune suppression. Herpetic infections are the classic example: ruxolitinib significantly increases herpes zoster reactivation risk (19). Prophylactic antivirals (e.g. acyclovir) are now recommended for patients on JAK inhibitors to mitigate this risk. Aside from infections, some ruxolitinib-treated patients report mild ocular irritation or redness, but data on incidence are limited. Other JAK inhibitors (fedratinib, pacritinib) are newer and haven’t yet been specifically associated with unique ocular issues, though vigilance is warranted.

#### BRAF inhibitors (dabrafenib, vemurafenib)

Dabrafenib, used in BRAF V600E-mutant melanoma, has been linked to ocular side effects, particularly when combined with MEK inhibitors. Reported adverse events include uveitis, cystoid macular edema, uveal effusion, and serous/exudative retinal detachment. The incidence of CSR is reported in up to 2% of patients, while uveitis has also been documented, though exact rates for the latter remain unspecified (36). Management typically involves temporary discontinuation of the offending agent and ophthalmologic evaluation, while corticosteroids should be avoided as they may exacerbate CSR. Vemurafenib, another BRAF inhibitor for metastatic melanoma, has ocular adverse effects in about 22% of patients. The most common issues are uveitis (4%), conjunctivitis (2.8%), and dry eyes (2%). These events are generally manageable without discontinuing therapy. However, rare cases of severe uveitis, such as Vogt-Koyanagi-Harada-like syndrome, have been reported, requiring more intensive management (37).

#### FLT3 inhibitors (midostaurin, gilteritinib)

FLT3-targeted agents used for AML generally lack a strong ocular toxicity signal. Midostaurin’s prescribing information notes eyelid edema (~3%) as an ocular adverse event, with rare reports of blurred vision or photophobia. Broader safety summaries have not identified higher-grade or structural ocular toxicity (20). Gilteritinib trials and post-marketing data describe infrequent visual changes (e.g., blurred vision, dry eye, or retinal hemorrhage, ~6–7%) and rare occurrences of differentiation syndrome that may include visual symptoms (21, 42).

#### Other TKIs/small-molecule inhibitors

A variety of other kinase inhibitors are used or in trials for hematologic cancers (e.g. PI3K inhibitors like idelalisib, BCL2 inhibitor venetoclax, MEK inhibitors in trials for certain leukemias). PI3K inhibitors (idelalisib, duvelisib) can cause immune-mediated side effects; ocular-specific data are sparse, but case reports note blurred vision or eye pain rarely, possibly related to drug-induced fevers or inflammation. Venetoclax is not a TKI but a targeted small molecule; sporadic cases of blurred vision have been reported (40), typically without detectable ocular pathology on examination. This effect may relate to transient hypotension or metabolic changes during tumor lysis and generally resolves spontaneously (43). Erdafitinib, an FGFR1–4 inhibitor approved for metastatic urothelial carcinoma, has been associated with notable ocular toxicities. According to the FDA label, erdafitinib is associated with ocular toxicities, including CSR and retinal pigment epithelial detachment (RPED), reported in 22% of patients with a median onset of 46 days (44). Management often involved dose interruptions or reductions, and in some cases, permanent discontinuation was necessary. Notably, 67% of patients who resumed therapy after interruption experienced recurrence or worsening of CSR (38). Additionally, a case report described bilateral multifocal serous retinal detachments associated with subretinal fluid accumulation and concurrent severe dry eye with unilateral peripheral ulcerative keratitis during erdafitinib therapy, which improved after drug discontinuation (45). This case highlights the diverse spectrum of ocular toxicities associated with targeted therapies, including uveal effusion, ocular surface disease, and immune-mediated inflammatory conditions such as PUK, and underscores the clinical significance of these distinct mechanisms, even when observed in single case reports. Lastly Crizotinib, targeting ALK and ROS1 in non-small cell lung cancer, frequently leads to visual disturbances. Approximately 63% of patients reported visual impairments, including photopsia, blurred vision, and vitreous floaters, typically within the first week of treatment. While most cases were mild (Grade 1), severe visual loss (Grade 4) was rare, occurring in 0.2% of patients (39). The underlying mechanism may involve abnormal signal processing in the retina. Proteasome inhibitors (bortezomib, carfilzomib, ixazomib) also intersect with this category of targeted drugs; their ocular effects are addressed in the “Other targeted agents” section below.

#### Mechanisms

The mechanisms behind TKI ocular toxicities vary by drug. For BCR-ABL inhibitors, inhibition of PDGFR and c-KIT in periorbital tissues is thought to cause local fluid retention and edema (6). The eyelid skin and orbital soft tissue are rich in these receptors; imatinib’s blockade of PDGF receptors leads to leaky vasculature and accumulation of glycosaminoglycans, manifesting as periocular swelling and conjunctival edema (6). The timing (often 1–2 months into therapy) supports a gradual tissue accumulation effect. MEK/ERK pathway inhibition (though relevant mostly to solid tumor drugs) is known to disrupt the retinal pigment epithelium and cause serious, or exudative, retinal detachment, sometimes accompanied by uveal effusion (46); interestingly, some TKIs like ponatinib that hit multiple kinases might also disturb retinal vasculature integrity via endothelial effects, contributing to RVO or hemorrhage (46). BTK inhibitors may cause uveitis through off-target immune modulation because BTK is expressed in myeloid cells and microglia; ibrutinib also inhibits ITK (important in T-cells) which could tilt immune responses and provoke ocular inflammation in susceptible individuals (17). The exact pathway is unclear, but the temporal association and improvement on stopping the drug implicates the drug as a trigger. JAK inhibitors reduce immune surveillance, providing a clear mechanism for viral reactivations like zoster (19). Some TKIs might also have direct toxic metabolites that accumulate in ocular tissues, but evidence is limited. Notably, imatinib has been detected in tear fluid, which could locally affect ocular surface cells over time (potentially explaining some dry eye complaints). Overall, off-target kinase inhibition, fluid retention, vascular effects, and immune effects are the main contributors to TKI-related ocular events.

#### Management

Most TKI-related ocular side effects can be managed conservatively or with adjunct therapy, without permanently discontinuing the TKI. For periorbital edema on imatinib, measures include cold compresses, dietary salt restriction, and diuretics if severe, along with patient reassurance, as the condition is typically non–vision-threatening and manageable with supportive care (47). If edema is very troublesome, switching to an alternate TKI (e.g. dasatinib or nilotinib) can be considered, as the edema tends to be most pronounced with imatinib. Conjunctival hemorrhages require no specific treatment and resolve spontaneously; lubricating drops can help if there is irritation. Blurred vision complaints should prompt a comprehensive ophthalmologic evaluation to determine the underlying cause and assess whether additional treatment or modification of therapy is warranted. Macular edema from TKIs (reported with imatinib and ponatinib) has been treated with topical, periocular, or intraocular steroids and sometimes temporary drug interruption, cases often improved, and patients resumed therapy at a reduced dose (15). For ponatinib, given its association with serious retinal vascular events, baseline and periodic dilated fundus examinations are recommended for patients with preexisting ophthalmic conditions or other ocular risk factors. If a patient on ponatinib develops retinal vein occlusion or severe vision loss, discontinuation is warranted and standard retinal treatments (anti-VEGF injections for macular edema, etc.) should be employed (48). Ibrutinib induced uveitis is managed with topical corticosteroids and cycloplegic drops; in reported cases, some patients were able to continue ibrutinib with uveitis treatment, while others required a pause until inflammation subsided (17). If severe or vision-threatening uveitis occurs, switching to an alternative BTK inhibitor might be an option (though cross-toxicity is not well documented). Dry eye symptoms from any TKI can be managed with artificial tears and ointments as needed. Additionally, because TKIs can cause ocular surface changes, contact lens users should practice good hygiene or temporarily avoid contact lens use to prevent keratitis. For JAK inhibitor patients, prevention is key: antiviral prophylaxis (acyclovir) is standard to prevent zoster, and any signs of eye infection should be treated aggressively with antivirals or antibiotics.

#### Outcomes

Generally, TKI-related ocular side effects are reversible and non-threatening. Patients’ visual acuity typically returns to baseline after appropriate management or dose adjustment. Periorbital edema can persist chronically but does not threaten vision; it often stabilizes or improves after the first few months on imatinib (47). Uveitis related to ibrutinib has resolved with therapy in reported cases, with patients regaining normal vision (17). The one area of caution is vascular occlusions; these can cause permanent vision loss if not recognized promptly. Thankfully, they are rare. Drug-related optic disc edema/papilledema has been described in case reports, for example, a recent published case described probable ponatinib-induced papilledema with objective improvement after acetazolamide (35). Because papilledema or optic disc edema can also result from leukemic infiltration or other disease-related causes, clinicians should investigate alternative etiologies (including neuroimaging and CSF analysis) rather than attributing vision loss solely to the TKI. In our review, we found no broad evidence of permanent bilateral blindness from any TKI; isolated unilateral losses have been reported due to specific events (RVO, etc.). Thus, with prudent monitoring, the prognosis for TKI related ocular events is good. Nonetheless, patient education about reporting visual symptoms is important, as timely intervention can prevent minor issues from escalating.

#### Evidence gaps

While numerous ocular adverse effects of TKIs have been noted, data are often from case reports or subset analyses of trials. There is a need for more systematic tracking of eye symptoms in clinical trials of TKIs; ophthalmic exams are not routinely included in oncology trials, so mild to moderate ocular toxicities might be underreported (6). Moreover, pediatric data on TKI ocular effects are minimal; children with CML on imatinib, for example, might experience similar side effects (some pediatric CML reports mention periorbital edema anecdotally), but no dedicated pediatric analysis exists. The long-term ocular effects of decades of TKI therapy (as CML patients now may take TKIs for life) are also unknown; e.g., could chronic imatinib use contribute to early cataract formation? Such questions remain unanswered. Finally, mechanism research is needed: understanding why certain patients get, say, ibrutinib-induced uveitis while most do not could reveal risk factors (perhaps an underlying autoimmune predisposition) and help guide prophylactic measures.

### Ocular toxicity with immune checkpoint inhibitors

ICIs, notably PD-1 inhibitors (e.g. nivolumab, pembrolizumab) and CTLA-4 inhibitor ipilimumab, are now standard in some hematologic contexts (for example, PD-1 blockers in relapsed/refractory Hodgkin lymphoma) and are being investigated in others. ICIs unleash T-cell activity against cancer, but can also trigger immune-related adverse events (irAEs) affecting virtually any organ. The eye is no exception, although ocular irAEs are relatively uncommon compared to effects on skin, colon, or endocrine organs. Large oncology registries estimate around 1% of patients on ICIs develop clinically significant ocular complications (12). Some newer studies suggest the incidence may be slightly higher (2–5%) when including milder manifestations (13, 14). A recent systematic review of 290 published ocular ICI cases found that ophthalmic toxicities, while rare, span a broad range of inflammatory conditions (12). Notably, ocular adverse events appear to be more frequent with nivolumab, pembrolizumab, and especially with the nivolumab and ipilimumab combination therapy compared to other ICI regimens (12). Both adults and children receiving ICIs (in pediatric trials) appear susceptible, though most data come from adult cancer patients (cutaneous melanoma being the most common malignancy in reported cases) (12).

The most frequently reported ocular toxicity from ICIs is uveitis. In the large case review, uveitis accounted for ~46% of reported ocular events (12). This typically presents as anterior uveitis/iritis (pain, redness, photophobia) and can be bilateral. Some cases progress to panuveitis involving the vitreous and choroid. Notably, uveitis was especially associated with melanoma patients on combination ICI therapy, possibly related to shared antigens (melanoma-associated antigens are present in the eye’s melanocytes, leading to a Vogt-Koyanagi-Harada-like syndrome in some). VKH-like uveitis often produces serous retinal detachments that are also described in safety reports as exudative retinal detachment and uveal effusion. We use the terms distinctly for clarity and indexing: serous (or exudative) retinal detachment describes subretinal fluid accumulation without a retinal tear, typically due to inflammatory or vascular leakage, whereas uveal effusion refers to choroidal or ciliary body thickening with suprachoroidal fluid that may secondarily cause retinal detachment. In checkpoint inhibitor–associated VKH-like disease, choroidal hyperpermeability and uveal effusion are the likely drivers of the serous (or exudative) detachment (49). The second most common category is neuro-ophthalmic disorders (~24% of cases) (12). This includes conditions like myasthenia gravis (an autoimmune neuromuscular junction disorder often manifesting with ptosis and double vision), optic neuritis (inflammation of the optic nerve, causing vision loss), and cranial nerve palsies affecting eye movements. ICI-induced myasthenia gravis frequently occurs with concurrent ICI-induced myositis; patients present with ptosis and ophthalmoplegia requiring urgent care. Conjunctivitis and dry eye disease related to ICIs are also reported, though in the literature they appear less frequently than uveitis. Keratitis and scleritis/episcleritis constitute about 10–11% of reported cases (12). For example, cases of peripheral ulcerative keratitis and corneal melts have occurred with ICIs, albeit rarely. Orbital inflammation (orbital pseudotumor) was noted in ~11% of the reviewed cases, this can cause painful proptosis, diplopia, blurred vision from ocular surface disease, and permanent vision loss from compressive optic neuropathy. Retinal effects (9% of cases) include autoimmune retinopathies and serous retinal detachments; one classical entity is ICI-associated birdshot chorioretinopathy (an autoimmune retinal condition). Additionally, ICIs can exacerbate pre-existing autoimmune eye conditions: for instance, a patient with quiescent Graves’ ophthalmopathy (thyroid eye disease; TED) might experience a flare on nivolumab.

In clinical trials of ICIs specifically, the overall incidence of any ocular side effect is usually reported around 1– 2%. Dry eye symptoms may be underreported but likely occur in a few percent of patients (some series cite up to 5–8% if patients are asked directly) (12). Importantly, severe ocular events (Grade 3–4, vision threatening) are quite rare (<1%). There have been isolated instances of permanent vision loss such as optic neuritis leading to optic atrophy, or melanoma-associated retinopathy (MAR) causing retinal dysfunction, which may occur as a paraneoplastic manifestation of the underlying malignancy rather than as an adverse effect of the treating agent. These cases remain exceptional, and most patients recover with appropriate therapy.

#### Mechanisms

The mechanisms of ICI-related ocular toxicities are grounded in autoimmunity. By blocking PD-1 or CTLA-4, ICIs remove inhibitory checkpoints on T-cells, which can then attack normal tissues expressing antigens that the immune system finds similar to tumor antigens or that were previously tolerated. The eye has many unique autoantigens (retinal proteins, uveal melanocytes, etc.) that can become targets. For example, CTLA-4 inhibitors like ipilimumab can prompt a T-cell attack on the uveal melanocytes, leading to uveitis, especially in melanoma patients (melanoma antigens cross-react with ocular melanocytes in Vogt-Koyanagi-Harada syndrome–like fashion) (50). PD-1 pathway blockade might incite Th1-mediated inflammation on the ocular surface, causing dry eye and keratitis analogous to Sjögren’s syndrome. Some events may represent a form of paraneoplastic syndrome unmasked by the immune system’s heightened state (51), for instance, cancer-associated retinopathy (where antibodies against tumor antigens cross-react with retinal cells) can emerge or worsen with immunotherapy. Additionally, immune checkpoints exist in the eye’s immune-privileged environment to maintain tolerance; blocking them can break ocular immune privilege, leading to inflammation. There is also a known association between ICIs and inflammatory cranial neuropathies (like optic neuritis) because unleashed T-cells can attack myelin or neuronal antigens in a manner similar to multiple sclerosis. Interestingly, the fact that neuro-ophthalmic events are often tied to lung cancer patients on anti–PD-1 (per the review data) suggests some tumor antigen might prime an attack on neuromuscular components (e.g. muscle acetylcholine receptors in myasthenia). In summary, ICI ocular toxicities are immune driven irAEs; essentially autoimmune diseases of the eye triggered by the therapy.

#### Timing

Ocular irAEs can occur at various points during ICI therapy, but many present within the first 3–6 months of treatment. Published series indicate a median onset around 8–12 weeks into therapy for uveitis and similar events (52). Myasthenia gravis tends to appear early (often after 2–3 ICI infusions, sometimes as part of a tri-syndrome with myositis and myocarditis). There are reports of very early onset (after the first dose) uveitis, as well as late-onset cases after a year of therapy, though late presentations are less common. If combination immunotherapy is used (e.g. ipilimumab + nivolumab), ocular toxicities may appear sooner and potentially with greater severity, analogous to other irAEs (12). It’s also observed that ocular irAEs can occur after cessation of therapy, during the immune reconstitution phase, for example, a patient might finish planned nivolumab and a month later develop uveitis as a delayed irAE. On the other hand, some ocular toxicities accompany systemic irAEs simultaneously (a patient with severe skin rash or hepatitis from ICI might concurrently develop eye inflammation). Thus, timing is variable, but the majority will manifest in the first few cycles of therapy. Continuous vigilance through the course of ICI treatment (and even for a few months after stopping) is advised.

#### Management

The management of ocular irAEs follows principles outlined in the recently published international Delphi consensus on ophthalmic immune-related adverse events (53), which established standardized definitions, grading criteria, and severity-based treatment algorithms. In line with these recommendations, effective management requires close collaboration between oncologists and ophthalmologists, with immunosuppression tailored to the severity of the ocular condition. For mild cases (e.g. mild dry eye or conjunctivitis), supportive care with artificial tears, lubricating ointments, and possibly topical anti-inflammatory drops (like low-dose steroid or cyclosporine drops for dry eye) may suffice, and ICI therapy can often be continued. For uveitis, topical corticosteroid eye drops (e.g. prednisolone acetate 1%) and cycloplegic drops (to relieve pain and prevent synechiae) are first-line. If uveitis is more than mild (≥Grade 2 or affecting vision), systemic corticosteroids are typically added (e.g. oral prednisone 0.5–1 mg/kg). In many cases of ICI-induced uveitis, checkpoint inhibitor therapy is held until the uveitis improves; if the uveitis is severe (Grade 3–4), permanent discontinuation of the ICI may be necessary. Immune-related optic neuritis or severe retinal inflammation is treated as an emergency – high-dose IV corticosteroids (e.g. methylprednisolone 1g/day for 3–5 days) are given to try to preserve vision. ICIs are discontinued in this scenario, and neurology consultation is obtained (since optic neuritis could be part of a broader demyelinating syndrome). Myasthenia gravis induced by ICIs (even though it manifests with ocular signs like ptosis) often requires IVIG or plasma exchange and systemic steroids, along with permanent ICI cessation, because it can be life-threatening. Scleritis or keratitis from ICIs is managed with topical steroids and sometimes systemic NSAIDs or steroids, similar to idiopathic cases. In refractory ocular inflammation, steroid-sparing immunosuppressants (such as infliximab for uveitis, or mycophenolate) have been used. Importantly, if an ocular irAE is isolated and mild, oncologists have successfully rechallenged patients with the immunotherapy after resolution, with close monitoring. Each decision to continue or stop ICIs is individualized, weighing cancer control against risk to vision (53).

#### Outcomes

The prognosis of ocular irAEs is generally favorable if recognized and treated early. In the majority of reported cases, patients recovered normal or near-normal vision. Uveitis typically responds to steroids, and vision returns to baseline in a few weeks (12). Notably, a large population-based cohort study from Kaiser Permanente Southern California quantified the real-world burden of ophthalmic irAEs across nearly 4.7 million patients (54), finding that the prevalence of uveitis and neuro-ophthalmic complications was significantly higher in patients with cancer than in those without cancer, and particularly elevated among melanoma patients receiving ICIs. The 1-year incidence of ICI-related uveitis was 1.2% in melanoma versus 0.2% in nonmelanoma cancers, highlighting that underlying malignancy type influences ocular irAE risk (54). Myasthenia gravis can result in chronic need for immunotherapy (the other kind: like pyridostigmine or IVIG), but if managed, patients’ ocular muscle function can improve. There have been cases of permanent optic nerve damage from optic neuritis when treatment was delayed; one patient in literature had persistent visual field defects after late recognition of checkpoint-induced optic neuritis. However, such outcomes are the exception. The key is prompt involvement of specialists: when treated, even serious conditions like Vogt-Koyanagi-Harada-like uveitis often resolved with high-dose steroids, with the detachments reattaching and vision improving over time. One study noted that about 80% of ICI-related uveitis cases had complete resolution, and 20% had partial resolution or chronic mild inflammation requiring ongoing topical therapy (55). Ocular side effects alone were rarely a cause of cancer therapy termination, usually, they coincided with other irAEs or were manageable enough to continue cancer treatment with adjustments. This is encouraging, as it suggests that with proper management, patients do not necessarily have to sacrifice cancer control for vision, or vice versa.

#### Gaps and recommendations

Despite being rare, ocular toxicities from ICIs illustrate the need for awareness. Oncologists should include vision changes in review of systems for patients on ICIs, and have a low threshold for ophthalmology referral, since early intervention is critical. Currently, there are no formal guidelines on baseline eye exams before ICI therapy; given the low incidence, universal screening isn’t cost-effective. However, population-based and large registry data indicate that patients with a prior history of uveitis or other ocular inflammatory disease have substantially higher recurrence rates after ICI initiation (54, 56, 57), supporting targeted baseline ophthalmic evaluation for these high-risk patients. The literature on pediatric ICI use (mostly experimental) and ocular side effects is nearly nonexistent, an area for future reporting as pediatric trials expand. A major gap is understanding risk factors: Why do only ~1–3% get these eye toxicities? Some hypotheses include the patient’s underlying tumor antigenicity (melanoma patients are overrepresented in uveitis cases) and genetic predispositions (HLA types associated with uveitis, for example). Research into biomarkers that predict irAEs (e.g. autoantibody development) could potentially identify patients at higher risk for ocular irAEs. Moreover, long-term follow-up of resolved ocular irAEs is needed; we need to know if any chronic ocular conditions persist (like glaucoma from steroid use, cataracts, etc., in these patients). In summary, ICIs can cause a diverse array of ocular autoimmune complications, and continued documentation and study of these events will help refine guidelines for monitoring and management.

### Ocular toxicity with other targeted agents (monoclonal antibodies, ADCs, and others)

Beyond CAR T, BiTes, TKIs, and ICIs, hematologic oncology employs numerous other targeted therapies, including monoclonal antibodies (mAbs), and antibody–drug conjugates (ADCs), as well as immunomodulatory drugs and proteasome inhibitors. Many of these have unique ocular side effect profiles listed below:

#### Monoclonal antibodies

Most monoclonal antibodies used in hematologic malignancies, such as rituximab (anti-CD20), alemtuzumab (anti-CD52), and daratumumab (anti-CD38), are not commonly associated with direct ocular toxicities. Rituximab has been linked to serum sickness–like reactions, which are immune complex-mediated and can occur days to weeks after infusion, particularly after the first dose. These reactions typically present with fever, rash, and arthralgia, though ocular involvement is rarely reported (58). Optic neuritis has been observed in patients receiving rituximab for neurologic autoimmune disorders such as myelin oligodendrocyte glycoprotein antibody-associated disease (MOG AD), but is exceedingly rare in cancer patients (59). Alemtuzumab has been associated with autoimmune thyroid disease, which in some cases can lead to TED, particularly in patients with multiple sclerosis (23). TED may cause symptoms like redness, dryness, and periorbital swelling, but alemtuzumab itself does not directly target ocular tissues. Daratumumab has not shown consistent ocular toxicity, although a case of choroidal effusion resulting in transient myopic shift and angle-closure glaucoma has been reported. This was likely due to the interaction of daratumumab with CD38 expressed on ocular tissues like the ciliary body (24). While immune complex deposition from chimeric antibodies could, in theory, lead to ocular inflammation such as microvasculitis, a consistent pattern of ocular vasculitis has not emerged across these agents in the literature.

#### Antibody–drug conjugates

ADCs consist of antibodies linked to cytotoxic payloads, allowing targeted delivery of chemotherapy to cancer cells. Some ADCs used in hematology have significant ocular toxicity issues:

##### Belantamab mafodotin (anti-BCMA ADC for multiple myeloma)

carries a microtubule inhibitor (MMAF) as its payload, and it has a well-documented ocular side effect: corneal epithelial toxicity. In clinical trials (DREAMM-2), around 71–77% of patients on belantamab developed corneal keratopathy characterized by epithelial changes (also called pseudomicrocysts) in the cornea (22). Patients experience symptoms like blurred vision, dry eye, and photophobia, and on exam have superficial punctate keratopathy or whorl-like epithelial patterns (60). This keratopathy can be severe (Grade 3–4 in ~45% of patients in trials) and has led to dose reductions or holds in the majority of patients (60). The mechanism is believed to be because corneal cells are particularly susceptible to the MMAF toxin. Notably, BCMA is not expressed in the cornea, indicating the effect is off-target. Management of belantamab keratopathy involves mandatory ophthalmologic exams prior to each dose (as per clinical protocols and current product labeling, with baseline and pre-dose exams) and withholding the drug until the slit lamp findings and symptoms improve above a set threshold. The keratopathy is felt to be reversible after discontinuation of the drug (median time to resolution around 3 months), but repeated cycles can cause recurrent injury. This example underscores how an effective cancer drug can be hampered by ocular side effects; belantamab achieved meaningful responses in myeloma, but ocular toxicity was dose-limiting in many. While other ADCs in hematology do not demonstrate equally dramatic ocular effects, the experience with belantamab and similar cases in solid tumors (e.g., some ADCs causing conjunctivitis or keratitis) still suggests that ophthalmic monitoring is prudent when introducing new ADCs. In the United States, marketing authorization was withdrawn after DREAMM-3 did not confirm clinical benefit, rather than primarily due to ocular safety. The European Commission approved belantamab combinations in July 2025 (61); an FDA decision on combinations is pending with a PDUFA date of October 23, 2025 (62).

##### Brentuximab vedotin (anti-CD30 ADC for Hodgkin lymphoma and T-cell lymphomas)

Brentuximab Vedotin carries the MMAE payload. While brentuximab’s main toxicities are peripheral neuropathy and cytopenias, corneal changes have been observed occasionally. ADCs with tubulin inhibitors (MMAE/MMAF) are known to cause ocular surface issues across different drugs (60). A pharmacovigilance analysis noted that ocular adverse effects are seen with many ADCs, and brentuximab was mentioned to have cases of keratopathy and dry eye (60). The incidence, however, is much lower than with belantamab. It is possible that some Hodgkin lymphoma patients on brentuximab experience mild dry eye or blurry vision, but this often goes unreported or attributed to other chemotherapies in combination. No formal ocular monitoring appears to be required for brentuximab, but clinicians should be aware of potential symptoms.

Gemtuzumab Ozogamicin (anti-CD33 ADC for AML) and Inotuzumab Ozogamicin (anti-CD22 ADC for ALL): Gentuzumab Ozogamicin carry a calicheamicin payload. Their prominent toxicity is hepatotoxicity (veno-occlusive disease), and ocular toxicities are not a known class effect for them (63). Thus, not all ADCs are ocular offenders; it depends on the payload.

##### Immunomodulatory drugs (IMiDs: thalidomide, lenalidomide, pomalidomide)

IMiDs are used in myeloma and some lymphomas. Their side effects include neuropathy, cytopenias, and thrombosis, but ocular effects are rarely emphasized (29). Some patients on IMiDs report blurred vision or visual disturbances, which could be multifactorial (e.g. lenalidomide can cause thyroid dysfunction indirectly, leading to dry eyes). There have been rare cases of papilledema in patients on high-dose thalidomide, which may possibly be related to anemia or clots rather than a direct drug effect. Overall, no consistent ocular toxicity pattern is seen with IMiDs, and any eye symptoms are usually mild and reversible. Regular eye exams are not required beyond routine care.

##### Proteasome inhibitors (bortezomib, carfilzomib, ixazomib)

Proteasome inhibitors are a backbone of multiple myeloma therapy and have some distinctive ocular effects. Bortezomib, in particular, has been associated with a high incidence of eyelid disorders. Up to 20–30% of patients on bortezomib develop chalazia (25). Patients often present with multiple recurrent chalazia and blepharitis (eyelid margin inflammation) after a few cycles of bortezomib. In a case series of 16 myeloma patients, 88% developed chalazia and 62% blepharitis; half had multiple episodes or multiple lesions concurrently (55). The median time to the first eyelid complication was around 3 months into therapy (25). The pathophysiology is not fully understood, but bortezomib’s effect on the immune system and skin appendages likely triggers meibomian gland inflammation and blockage, akin to acneiform rashes seen with some targeted agents (26). Bortezomib may induce a pro-inflammatory state (patients often get skin rashes and shingles as well), which could explain the eyelid issues (27). Management involves conservative local therapy: warm compresses, lid hygiene, antibiotic/steroid ointments, and in some cases minor surgical incisions for chalazia. Notably, bortezomib-induced chalazia can be quite refractory to standard treatments and tend to recur until bortezomib is reduced or stopped (25, 26). One small study found that prophylactic anti-inflammatory eye drops (like ketotifen, a mast-cell stabilizer) may reduce the frequency of new chalazia in these patients (27). Carfilzomib, a related IV proteasome inhibitor, seems to have a lower incidence of these issues (only one case in the series above showed chalazia occurring on carfilzomib after prior bortezomib) (25). Ixazomib (oral proteasome inhibitor) in trials showed a surprisingly high rate of ocular adverse events. In one study, 32% of patients on ixazomib reported some ocular side effect, most commonly blurred vision (7%) (28). This might be due to the ease of reporting in a trial setting or possibly an off-target effect of ixazomib on the visual pathway, though the mechanism is unclear. Regardless, proteasome inhibitors highlight the need to consider even seemingly trivial symptoms like styes as drug-related. While chalazia do not threaten vision, patients find multiple chalazia irritating and cosmetically concerning. Oncologists should ask about eye comfort in myeloma patients and involve ophthalmology for persistent chalazia; in refractory cases, to balance safety and efficacy, switching from bortezomib to an alternative agent can be considered if feasible.

#### Major gaps

Many of these other agents have come to market in the last decade, and their full spectrum of ocular effects is still being elucidated. Extensive ocular monitoring in belantamab mafodotin trials brought detailed information about its toxicity profile to light, whereas other drugs without mandated eye exams might have hidden effects. Real-world data collection (e.g. post-marketing surveillance) is crucial to catch less obvious and rarer ocular adverse effects for agents like bispecific T-cell engagers, bispecific antibodies, and novel mAbs. Pediatric considerations are also underreported here; for children receiving anti-CD19 bispecifics or novel ADCs, we have virtually no published data on their ocular side effects, which may differ or be harder to assess in kids. Furthermore, the pathophysiology of some of these effects (like bortezomib chalazia or ixazomib blurred vision) is not well understood; clinical and animal model research into whether these are immune-mediated or direct cellular effects could help in developing preventive strategies. Another gap is long-term follow-up: do patients with belantamab-induced keratopathy have any residual corneal issues a year after stopping therapy? How do we rehabilitate vision in those who had severe keratopathy? These questions need longitudinal studies. Lastly, as new targeted agents emerge (e.g. cereblon E3 ligase modulators in myeloma and chimeric antigen receptor NK-cell therapies, etc.), it will be important to systematically monitor for ocular AEs from the outset, learning from the lessons of prior therapies.

## Discussion

In this review, we synthesized data on ocular toxicities associated with a broad range of modern hematologic cancer therapies. Despite the heterogeneity of agents, from cell therapies to small molecules, a unifying theme is that ocular side effects, while generally infrequent, can occur across virtually all these treatment modalities. Our review highlights that incidence rates vary widely by therapy class and specific agents. For example, immune checkpoint inhibitors cause ocular immune-related adverse events (ocular irAEs) in roughly 1–4% of patients (12). While most CAR T-cell recipients (~98%) will not have ocular issues, a small subset (1–2%) may develop serious neuro-ophthalmic complications (3). TKIs have a spectrum from very common mild effects (imatinib periorbital edema ~60%) to rare severe events (ponatinib retinal vascular occlusion ~2%) (34). Additionally, ADCs like belantamab can affect nearly every treated patient’s eyes, with keratopathy showing in ~70–75% of patients (24). Notably, pediatric specific data are limited, but we expect similar toxicity profiles in children based on the mechanisms involved.

Pathophysiologic mechanisms often mirror the therapeutic action: immunotherapies (CAR T, ICIs, bispecifics) tend to cause inflammatory or immune-mediated ocular conditions (uveitis, optic neuritis, etc.), whereas targeted drugs that inhibit signaling pathways (TKIs) cause ocular effects related to those pathways’ roles in ocular tissues (e.g. PDGFR inhibition causing edema, or off-target kinase inhibition causing epithelial dysfunction). Some agents cause ocular harm via direct toxicity to ocular cells, with the clearest example being ADC keratopathy (22). Others exert indirect effects: JAK inhibitors don’t harm the eye directly but predispose to infections like zoster that can involve the eye (19). Understanding these mechanisms is not merely academic; it guides management. For instance, knowing that an ocular issue is immune-related (like ICI-induced uveitis) prompts immunosuppressive therapy, whereas knowing it’s directly drug–related (like ADC keratopathy) leads to dose adjustment and local supportive care rather than systemic immunosuppression.

Clinically, the types of ocular toxicities observed cover a large swath of ophthalmology: surface diseases (dry eye, conjunctivitis, chalazia), anterior segment inflammation (anterior uveitis, scleritis), posterior segment issues (exudative retinal detachments or small serous retinal detachments, retinal hemorrhages, posterior uveitis), optic nerve edema, cranial nerve palsies, and more. Fortunately, the majority are Grade 1–2 in severity (e.g. mild dry eye or transient blurred vision). However, a significant minority are Grade ≥3 (e.g. severe keratitis with vision loss, or uveitis requiring systemic steroids). The timeline of onset is usually within the first few cycles or months of therapy for most agents, emphasizing the need for early monitoring. For chronic oral therapies (like TKIs for CML), some effects like periorbital edema are chronic but manageable, whereas rarer events like cataracts or glaucoma might only be noted on long-term follow-up (not clearly seen in shorter study timeframes, but something to watch for as patients age on these drugs).

In terms of outcomes, the encouraging finding is that most therapy-associated ocular toxicities are reversible with appropriate intervention. Our review did not find evidence of high rates of permanent blindness solely attributable to these treatments. Cases of irreversible damage were isolated (e.g. a missed immune-mediated optic neuritis or a severe retinal artery occlusion). This suggests that with vigilant care, patients can avoid serious outcomes. Management strategies have evolved for many of these toxicities: for instance, standardized ophthalmic monitoring and dose modification guidelines for belantamab mafodotin have been in place due to FDA mandates; checkpoint inhibitor-related uveitis is now part of oncologists’ differential when a patient has eye pain, and prompt steroid treatment can preserve vision; and simple measures like prophylactic antiviral medication in ruxolitinib patients can prevent an HZO that might scar the cornea. Interdisciplinary care is a recurring theme, the need for oncologists, hematologists, and ophthalmologists (sometimes ocular oncologists, neuro-ophthalmologists, or uveitis specialists) to collaborate. In many comprehensive cancer centers, supportive care pathways now include ophthalmology input for patients on certain trials or therapies (especially ADCs and ICIs).

### Implications for practice

Oncologists should be aware of the potential ocular side effects of the agents they prescribe and inform patients accordingly. Even when uncommon, patient education can lead to less psychological stress on patients, as well as, earlier reporting of symptoms. For example, a CML patient on imatinib who knows that periorbital swelling is an expected side effect may be less alarmed and more compliant. A patient on an ICI who is told about possible eye inflammation will likely seek care promptly if vision changes occur, rather than waiting. It is not unreasonable, in select cases, that baseline ophthalmologic exams be considered for therapies with a known high incidence of ocular events. Routine screening during therapy is generally indicated only for select agents (the prime example being belantamab, with mandated eye exams every 3 weeks). For most others, symptom triggered evaluation is appropriate.

Major research gaps identified include: (1) A lack of a deep prospective dataset on ocular toxicities – future trials of novel agents should incorporate ophthalmic assessments when signals emerge. (2) Minimal information on pediatric patients as children might experience toxicities differently. (3) The need to delineate mechanistic pathways – e.g., why do certain patients on ibrutinib get uveitis? Are there predictive biomarkers (such as autoantibodies or particular HLA types)? Research into the immune profiles of those who develop ocular irAEs vs those who do not could shed light here. (4) Long-term outcomes: As therapies like CAR T-cell are still relatively new, we do not know if there are late ocular effects such as accelerated aging changes or secondary autoimmunity. Additionally, the cumulative effect on vision of sequential therapies (many patients receive multiple lines of therapy) is unknown – a patient might receive chemotherapy, then a TKI, then an ICI, etc., each with potential subclinical ocular impacts that could sum up. (5) Management strategies need refinement – for some toxicities like bortezomib induced chalazia, we lack evidence-based prevention or treatment beyond case series; controlled studies of prophylactic measures (like the ketotifen drop idea) would be valuable. Similarly, for belantamab, alternative dosing schedules are being researched to mitigate keratopathy while retaining efficacy (22).

Our review has some limitations in that the data we compiled often came from heterogeneous sources (case reports and clinical trials) which have different reporting biases. Incidence figures from trials may under-report minor ocular symptoms, whereas pharmacovigilance databases lack accurate denominators to calculate true incidence. We attempted to emphasize higher-level evidence (e.g. systematic reviews, prospective studies) when available, but for rare toxicities, anecdotal evidence was sometimes all that existed. Another limitation is that the literature up to 2025 may not capture evolving practices (for example, the withdrawal and potential re-introduction of belantamab with modified protocols). Despite these, the approach we used helps ensure we covered the breadth of known information up to the present.

## Conclusion

Ocular toxicities in hematologic cancer therapy represent an intersection of oncology and ophthalmology that clinicians, patients, and care-givers must navigate as targeted and immune-based treatments proliferate. While most patients will not experience serious eye issues, a small but important subset will – and for those, early recognition and management is paramount to optimize patient management. We have detailed the incidence, mechanisms, types, timing, and management of these toxicities for CAR T-cell therapies, TKIs, ICIs, mAbs, bispecifics, ADCs, and IMiDs, and proteasome inhibitors. With increasing survival in hematologic malignancies, survivorship care should include attention to ocular health, especially in those on long-term targeted treatments. Close collaboration between hematologist-oncologists and eye care specialists, patient education, and further research into preventive and therapeutic strategies for ocular AEs will together improve outcomes. Future studies should aim to fill current knowledge gaps, particularly through prospective monitoring and global reporting of ocular events. By doing so, we can hopefully mitigate ocular risks while harnessing the full benefit of these life-saving therapies.

## Glossary

ADC: Antibody–Drug Conjugate
ALL: Acute Lymphoblastic Leukemia
AML: Acute Myeloid Leukemia
BCMA: B-cell Maturation Antigen
BiTE: Bispecific T-cell Engager
CAR T: Chimeric Antigen Receptor T-cell
CLL: Chronic Lymphocytic Leukemia
CML: Chronic Myeloid Leukemia
CN: Cranial Nerve
CRS: Cytokine Release Syndrome
CTLA-4: Cytotoxic T-Lymphocyte–Associated Antigen 4
FDA: Food and Drug Administration
FGFR: Fibroblast Growth Factor Receptor
FLT3: Fms-like Tyrosine Kinase 3
GA: Geographic Atrophy
GIST: Gastrointestinal Stromal Tumor
HZO: Herpes Zoster Ophthalmicus
ICANS: Immune Effector Cell–Associated Neurotoxicity Syndrome
ICI: Immune Checkpoint Inhibitor
irAE: Immune-related Adverse Event
IVIG: Intravenous Immunoglobulin
JAK: Janus Kinase
MAC: Membrane Attack Complex
MMAF: Monomethyl Auristatin F
NSCLC: Non–Small Cell Lung Cancer
PD-1: Programmed Cell Death Protein 1
PDGFR: Platelet-Derived Growth Factor Receptor
PRISMA: Preferred Reporting Items for Systematic Reviews and Meta-Analyses
PUK: Peripheral Ulcerative Keratitis
ROP: Retinopathy of Prematurity
RPED: Retinal Pigment Epithelial Detachment
TKI: Tyrosine Kinase Inhibitor
VEGFR: Vascular Endothelial Growth Factor Receptor
VKH: Vogt-Koyanagi-Harada (syndrome)
